# A system for success: *BMC Systems Biology*, a new open access journal

**DOI:** 10.1186/1752-0509-1-41

**Published:** 2007-09-04

**Authors:** Matt J Hodgkinson, Penelope A Webb

**Affiliations:** 1 BioMed Central, Middlesex House, 34-42 Cleveland St., London, W1T 4LB, UK

## Abstract

*BMC Systems Biology *is the first open access journal spanning the growing field of systems biology from molecules up to ecosystems. The journal has launched as more and more institutes are founded that are similarly dedicated to this new approach. *BMC Systems Biology *builds on the ongoing success of the *BMC *series, providing a venue for all sound research in the systems-level analysis of biology.

## The *BMC *series – building on success

When the open access publisher BioMed Central launched the *BMC *series of journals in May 2000, it provided a venue for open access to research covering a wide range of research fields in biology and medicine [1] and the imperative to allow the free flow of scientific ideas [2]. The *BMC *series was one initiative from BioMed Central in response to recognizing that the quantity of results and data being generated was too much for conventional systems of publication to deal with. We needed a system that was open and allowed data, results, and interpretations to flow freely, be checked and stamped with authority, but also to be freely mined using computational tools.

The aim was to create a resource of scientific research that was freely available online, including for download and reuse – that is, open access. Open Access is more than just being free online – it also means being permanently archived in a public and accessible archive, and being freely distributable and reusable [3].

The *BMC *series has met that aim, and has proven to be highly successful both when measured by quantity – submissions have roughly doubled every 18 months, interestingly very much in line with Moore's law for computing [4] – and when measured by quality. The Impact Factor from Thomson Scientific (ISI) [5], although much criticised [6,7], is the most widely used metric for the "importance" of the results published in a journal [8]. Judging by the number of journals in the *BMC *series that have received good Impact Factors, such as *BMC Bioinformatics *[9], *BMC Cancer*, *BMC Evolutionary Biology *and *BMC Genomics *[10,11], the research published in these journals is certainly proving to be important and citable. Together with the other journals published by BioMed Central such as *Breast Cancer Research*, *Genome Biology *and *Journal of Biology*, the *BMC *series has proven that open access works.

We don't, however, want to rest on our laurels. In the last few years we have launched new *BMC *journals to address gaps in the series' coverage. *BMC Biology *and *BMC Medicine *launched at the end of 2003 to provide an outlet for high quality research of broad interest beyond a single discipline [12]. In response to a growing number of enquiries from veterinarians looking for an open access journal in their field, we also launched *BMC Veterinary Research *in 2005 [13].

## *BMC Systems Biology *– a new journal, in a new field

There has been another gap that has become evident in the biology journals. Studies were beginning to appear that seemed too expansive to fit the scope of our existing journals, encompassing but not contained within bioinformatics or genomics, nor biochemistry or physiology. That gap was systems biology, and we've now filled it.

**Figure 1:**
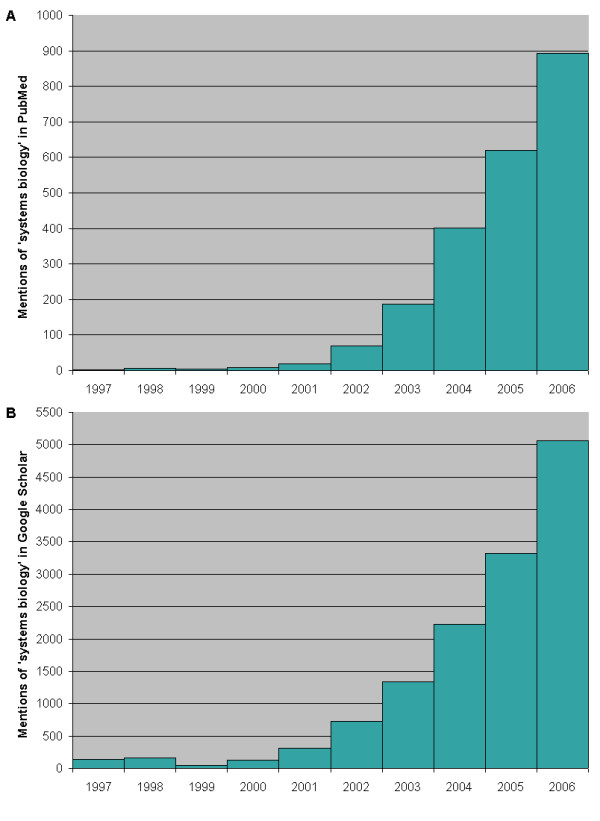
**The growth of the use of the term 'systems biology' over the last ten years**. **(a) **Number of references to 'systems biology' in PubMed abstracts per year since 1997. **(b) **Number of references to 'systems biology' in full text articles in Google Scholar per year since 1997.

There has been a rapid growth in the use of the term 'systems biology' in the literature as this new field emerges (see Figure 1). The field is in fact truly 'emergent' (a popular concept in systems biology [14]), arising as it does out of a combination of high-throughput techniques (epitomised by the microarray) along with the mathematical modelling that is made possible by computing power that would once have been housed within aircraft hangers and now sits upon a desktop.

## So what is it?

Systems biology has been defined many times [15-17]. However, the essence of systems biology is probably encapsulated within several concepts:

• Viewing biological systems as a **whole**, rather than solely in terms of their component parts;

• Mathematical **modelling**;

• **Iterative **analysis, with experimental data informing models, which in turn refine the experiments;

• Moving between and **integrating **systems at different scales – from atoms up to ecosystems;

• **Interdisciplinary **collaboration between researchers from diverse subject areas, both within biology (including physiologists, developmental biologists, evolutionary scientists, neuroscientists, cell biologists, genomicists to name but some), and beyond biology (drawing in mathematicians, physicists, computer scientists, and social scientists).

Systems biology is a new way of approaching the investigation of biology. Dedicated systems biology institutes have sprung up all over the world: pioneering Japanese and American institutes (The Systems Biology Institute in Tokyo [18], and the Institute for Systems Biology in Seattle [19], both founded in 2000), are now being joined by fledging Systems Biology institutes such as the first new department at Harvard for two decades [20], and the new BBSRC-funded centres for integrative systems biology recently established in Edinburgh, London, Manchester, Newcastle, Nottingham and Oxford [21].

## The story so far

The first articles published in *BMC Systems Biology *include work from Editorial Board member Douglas Lauffenburger concerning the use of decision trees to predict of the behaviour of fibroblasts [22], the identification of functional modules from Ron Shamir [23], and the latest software for analyzing metabolic, regulatory and signalling networks from Ernst Gilles [24]. Our international Editorial Board has provided vital support and guidance to ensure the rigorous review of all submitted manuscripts [25]. Between them, the first ten articles were accessed over 7,000 times in the first month of publication, and the ten most viewed articles have together been viewed by more than 20,000 readers [26].

## Conclusion

If you are a researcher exploring this new frontier of biology, we encourage you to consider submitting to *BMC Systems Biology*, even if you do not think of yourself as a 'system biologist'. You may be a neuroscientist, a physiologist or an ecologist, but if you are applying the key features of modelling and integration, we want to hear from you. While the focus of the journal is upon original research, we are also keen to publish commentaries that explore the expanding boundaries of the field of systems biology and suggest new opportunities for collaboration between disciplines, such as Nicolas Le Novère's exploration of the journey towards a systems biology of neuroscience over the last half century [27].

Nicholas Rajewsky, a member of our Editorial Board, said in summing up the launch of our new journal, "*The *BMC *journals have already proven to be very attractive to many scientists and readers. These journals provide open access, do not restrict manuscript length, and do not insist on publishing only 'major' findings, that is, they do not force scientists to strive for 'sensations'. Especially in a new field, such as systems biology, it is necessary to work on solid methodology development. Therefore*, BMC Systems Biology *seems especially suited to become an important and high impact journal" *[28]. We couldn't agree more.

## References

[B1] The *BMC *series. http://www.biomedcentral.com/info/authors/bmcseries.

[B2] Fletcher G Averting the crisis in medical publishing – open access journals. http://www.biomedcentral.com/html/info/about/FletcherHOITI.pdf.

[B3] Suber P A very brief introduction to open access. http://www.earlham.edu/~peters/fos/brief.htm.

[B4] Moore's Law. http://www.intel.com/technology/mooreslaw/index.htm.

[B5] The ISI Impact Factor. http://scientific.thomson.com/free/essays/journalcitationreports/impactfactor/.

[B6] Seglen PO (1997). Why the impact factor of journals should not be used for evaluating research. BMJ.

[B7] Monastersky R (2005). The Number That's Devouring Science. Chronicle of Higher Education.

[B8] Bollen J, Rodriguez MA, Van de Sompel H (2006). Journal status. ArXiv:csDL/0601030.

[B9] Cockerill M (2005). *BMC Bioinformatics *comes of age. BMC Bioinformatics.

[B10] Impressive new Impact Factors for BioMed Central's open-access journals. http://www.biomedcentral.com/info/about/pr-releases?pr=20060620b.

[B11] BioMed Central Impact Factor FAQ. http://www.biomedcentral.com/info/about/faq?name=impactfactor.

[B12] New Open Access choice for authors. Open Access Now.

[B13] Launch of *BMC Veterinary Research*. http://www.biomedcentral.com/info/about/pr-releases?pr=20050601.

[B14] Bhalla US, Iyengar R (1999). Emergent properties of networks of biological signaling pathways. Science.

[B15] A glossary for Systems Biology. http://www.sysbio.de/projects/glossary/Systems_Biology.shtml.

[B16] Douglas Kell's group at the Manchester Centre for Integrative Systems Biology. http://dbkgroup.org/sysbio.htm.

[B17] Kitano H (2002). Systems Biology: A Brief Overview. Science.

[B18] The Systems Biology Institute. http://www.sbi.jp/.

[B19] Institute for Systems Biology. http://www.systemsbiology.org/.

[B20] Department of Systems Biology, Harvard Medical School. http://sysbio.med.harvard.edu/.

[B21] BBSRC - The science we support. Systems Biology: Modelling, Simulation and Experimental Validation. http://www.bbsrc.ac.uk/science/areas/ebs/themes/main_sysbio.html.

[B22] Kharait S, Hautaniemi S, Wu S, Iwabu A, Lauffenburger DA, Wells A (2007). Decision tree modeling predicts effects of inhibiting contractility signaling on cell motility. BMC Systems Biology.

[B23] Ulitsky I, Shamir R (2007). Identification of functional modules using network topology and high-throughput data. BMC Systems Biology.

[B24] Klamt S, Saez-Rodriguez J, Gilles ED (2007). Structural and functional analysis of cellular networks with CellNetAnalyzer. BMC Systems Biology.

[B25] *BMC Systems Biology *Editorial Board. http://www.biomedcentral.com/bmcsystbiol/edboard/.

[B26] *BMC Systems Biology *most viewed articles. http://www.biomedcentral.com/bmcsystbiol/mostviewedalltime/.

[B27] Le Novère N (2007). The long journey to a Systems Biology of neuronal function. BMC Systems Biology.

[B28] BioMed Central launches *BMC Systems Biology*, a new open access journal. http://www.biomedcentral.com/info/about/pr-releases?pr=20070108.

